# Impact of Sodium Dichloroacetate Alone and in Combination Therapies on Lung Tumor Growth and Metastasis

**DOI:** 10.3390/ijms222212553

**Published:** 2021-11-21

**Authors:** Aya Al-Azawi, Shahrazad Sulaiman, Kholoud Arafat, Javed Yasin, Abderrahim Nemmar, Samir Attoub

**Affiliations:** 1 Department of Pharmacology & Therapeutics, College of Medicine & Health Sciences, United Arab Emirates University, Al-Ain 17666, United Arab Emirates; 201870001@uaeu.ac.ae (A.A.-A.); sharazadjeffy@uaeu.ac.ae (S.S.); kholoud.arafat@uaeu.ac.ae (K.A.); 2 Department of Medicine, College of Medicine & Health Sciences, United Arab Emirates University, Al-Ain 17666, United Arab Emirates; javed.yasin@uaeu.ac.ae; 3 Department of Physiology, College of Medicine & Health Sciences, United Arab Emirates University, Al-Ain 17666, United Arab Emirates; anemmar@uaeu.ac.ae; 4 Zayed Center for Health Sciences, United Arab Emirates University, Al-Ain 17666, United Arab Emirates; 5 Institut National de la Santé et de la Recherche Médicale (INSERM), 75013 Paris, France

**Keywords:** lung cancer, dichloroacetate, pyruvate dehydrogenase kinase inhibitor, tumor growth, angiogenesis, gefitinib, erlotinib

## Abstract

Metabolic reprogramming has been recognized as an essential emerging cancer hallmark. Dichloroacetate (DCA), an inhibitor of pyruvate dehydrogenase kinase (PDK), has been reported to have anti-cancer effects by reversing tumor-associated glycolysis. This study was performed to explore the anti-cancer potential of DCA in lung cancer alone and in combination with chemo- and targeted therapies using two non-small cell lung cancer (NSCLC) cell lines, namely, A549 and LNM35. DCA markedly caused a concentration- and time-dependent decrease in the viability and colony growth of A549 and LNM35 cells in vitro. DCA also reduced the growth of tumor xenografts in both a chick embryo chorioallantoic membrane and nude mice models in vivo. Furthermore, DCA decreased the angiogenic capacity of human umbilical vein endothelial cells in vitro. On the other hand, DCA did not inhibit the in vitro cellular migration and invasion and the in vivo incidence and growth of axillary lymph nodes metastases in nude mice. Treatment with DCA did not show any toxicity in chick embryos and nude mice. Finally, we demonstrated that DCA significantly enhanced the anti-cancer effect of cisplatin in LNM35. In addition, the combination of DCA with gefitinib or erlotinib leads to additive effects on the inhibition of LNM35 colony growth after seven days of treatment and to synergistic effects on the inhibition of A549 colony growth after 14 days of treatment. Collectively, this study demonstrates that DCA is a safe and promising therapeutic agent for lung cancer.

## 1. Introduction

Lung cancer is the second most commonly occurring cancer, with the highest mortality rate worldwide, accounting for 2.2 million cases and 1.8 million deaths in 2020. Incidence and mortality are projected to continue to rise by approximately 60%, to an estimated 3.6 million and 3 million, respectively, in 2040 [1]. Most lung cancer cases are NSCLC, accounting for 80–85% of all lung cancer cases [2]. The development of targeted and immunotherapies has revolutionized the treatment of NSCLC. However, side effects, resistance and efficacity in a small therapeutically sensitive group of patients create inequalities in access to such agents [3,4,5]. Therefore, this underscores the need for safer and more efficacious agents.

Metabolic reprogramming is one of the cancer hallmarks that has been a promising target for the development of effective therapeutic approaches [6]. Compared to the normal cells that rely mainly on mitochondrial oxidative phosphorylation (OXPHOS) under aerobic conditions, cancer cells deviate from this normal metabolic phenotype by relying mainly on cytosolic glycolysis and lactic fermentation, even in the presence of oxygen, to meet the needs of high proliferation [7]. This phenomenon is known as the Warburg effect, which has been exploited as a therapeutic target to inhibit tumor growth [8]. PDK is among the essential enzymes controlling glycolysis and OXPHOS [9]. It shut down the mitochondrial OXPHOS by phosphorylating and inhibiting pyruvate dehydrogenase (PDH), a key enzyme catalyzing the oxidative conversion of pyruvate into acetyl coenzyme A in mitochondria [10]. 

DCA is a small-molecular-weight drug that was used in lactic acidosis, congenital mitochondrial defects and diabetes [11]. Interestingly, DCA showed an ability to shift the tumor metabolism from cytosolic aerobic glycolysis to mitochondrial OXPHOS by inhibiting PDK and enhancing the activity of PDH [12]. Hence, DCA has been reported to have anti-cancer effects by increasing the efflux of cytochrome c and other apoptotic-inducing factors and the upregulation of ROS levels with consequent cancer cell death [11,13,14,15]. However, in clinical investigations, the safety profile of DCA was a concern. Even so, Garon et al. (2014), who conducted a clinical trial with DCA on lung cancer patients, concluded that: “in the absence of a larger controlled trial, firm conclusions regarding the association between the patient’s adverse events and DCA are unclear”. They recommended that DCA should be considered with platinum-based chemotherapy in hypoxic tumors rather than as a single agent in advanced non-small-cell lung cancer [16]. DCA is believed to be a potent molecule that warrants further investigation of its anti-cancer potential on NSCLC.

This study aimed to investigate the impact of DCA on NSCLC cellular viability, colony growth, and cellular migration and invasion in vitro, in addition to tumor growth, metastasis and toxicity in vivo. In addition, DCA’s direct impact on angiogenesis was assessed in vitro. Furthermore, we investigated the effect of DCA in combination therapies with chemotherapy and the first generation of EGFR tyrosine kinase inhibitors (EGFR-TKi). 

## 2. Results

### 2.1. Effect of DCA on Cellular Viability and Colony Growth of NSCLC Cell Lines

**Figure 1:**
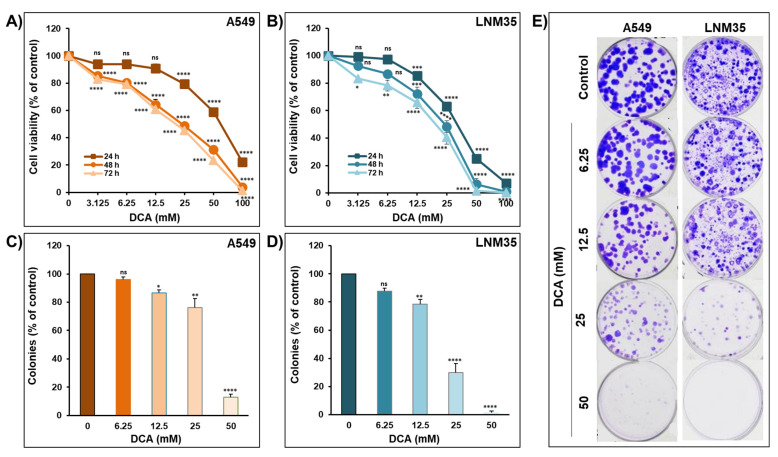
Effect of DCA on NSCLC cells’ viability and colony growth. Exponentially growing A549 (**A**) and LNM35 (**B**) cancer cells were incubated in the absence or presence of increasing concentrations of DCA (3.125–100 mM) for 24, 48 and 72 h. Cellular viability was assessed as described in the Materials and Methods. Experiments were repeated at least three times. Shapes represent means, bars represent S.E.M. A549 (**C**) and LNM35 (**D**) cancer cells were grown for 7 days to form colonies that were treated with different concentrations of DCA (6.25–50 mM) for 7 days after which colonies were fixed, stained and counted as described in the Materials and Methods. (**E**) Representative pictures of the control and DCA-treated colonies are shown for A549 and LNM35 cancer cells. Results are presented as percent colonies (mean ± S.E.M.) of treated cells compared to control. * Significantly different at <0.05. ** Significantly different at <0.01. *** Significantly different at <0.001. **** Significantly different at <0.0001. ns—non-significant.

The effect of increasing the concentration of DCA (3.125–100 mM) was investigated on two NSCLC cell lines, namely, A549 and LNM35. As shown in Figure 1, DCA reduced the viability of A549 (Figure 1A) and LNM35 (Figure 1B) in a concentration- and time-dependent manner. The half-maximal inhibitory concentration (IC_50_) of DCA at 48 h is approximately 25 mM for both cell lines. 

For further assessment of the anti-cancer effect of DCA, its impact was investigated on the growth of pre-formed colonies of A549 and LNM35 cell lines. Toward this, both cell lines were grown at a specific density for 1 week to form colonies and then treated with an increasing concentration of DCA for 1 week. As shown in Figure 1, DCA caused a concentration-dependent reduction in the number of colonies for both cell lines, with a higher sensitivity shown in LNM35 colonies (Figure 1D,E) compared to A549 colonies (Figure 1C,E). These results confirm the anti-cancer effect of DCA in vitro.

### 2.2. Effect of DCA on the Growth of NSCLC Tumor Xenografts in a Chick Embryo CAM and Nude Mice In Vivo

**Figure 2:**
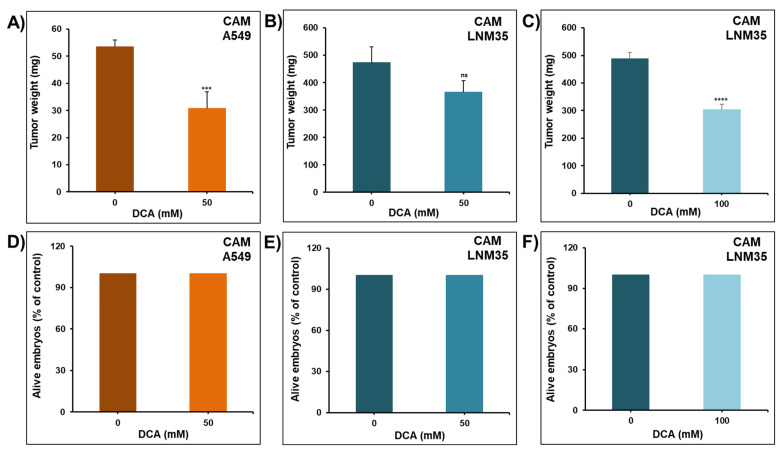
Effect of DCA on the growth of A549 and LNM35 tumor xenografts in the chick embryo CAM in vivo. (**A**) Tumor weight of A549 cancer cells xenografted on the CAM at a density of 1 million cells after treatment with drug vehicle (0.9% NaCl) or DCA (50 mM) for 1 week. (**B**,**C**) Tumor weight of LNM35 cancer cells xenografted on the CAM at a density of 0.3 million cells after treatment with drug vehicle (0.9% NaCl) or DCA (50 mM and 100 mM). (**D**) Percentage of alive embryos in the control and DCA-treated A549 xenografts. (**E**,**F**) Percentage of alive embryos in the control and DCA-treated LNM35 xenografts. Columns are means; bars are S.E.M. *** Significantly different at <0.001. **** Significantly different at <0.0001. ns—non-significant.

To confirm the pharmacological relevance of our in vitro results, the anti-cancer effect of DCA was evaluated in vivo using a chick embryo CAM assay. A549 and LNM35 xenografted tumors on the CAM were treated with 50 mM of DCA every 48 h for 1 week. At E17, tumors were recalled from the upper CAM and weighed. As observed in Figure 2, 50 mM of DCA significantly reduced the growth of A549 tumor xenografts by approximately 40% (Figure 2A), while it did not show a significant reduction in the growth of LNM35 tumor xenografts (Figure 2B). Therefore, 100 mM of DCA was investigated on LNM35 tumor xenografts and it significantly reduced the growth by approximately 40% (Figure 2C). Toxicity was also assessed by comparing the percentage of alive embryos in the control and DCA-treated groups. At E17, DCA showed no cytotoxicity as the percentage of the alive embryos was similar with the control and DCA treatments (Figure 2D–F).

**Figure 3:**
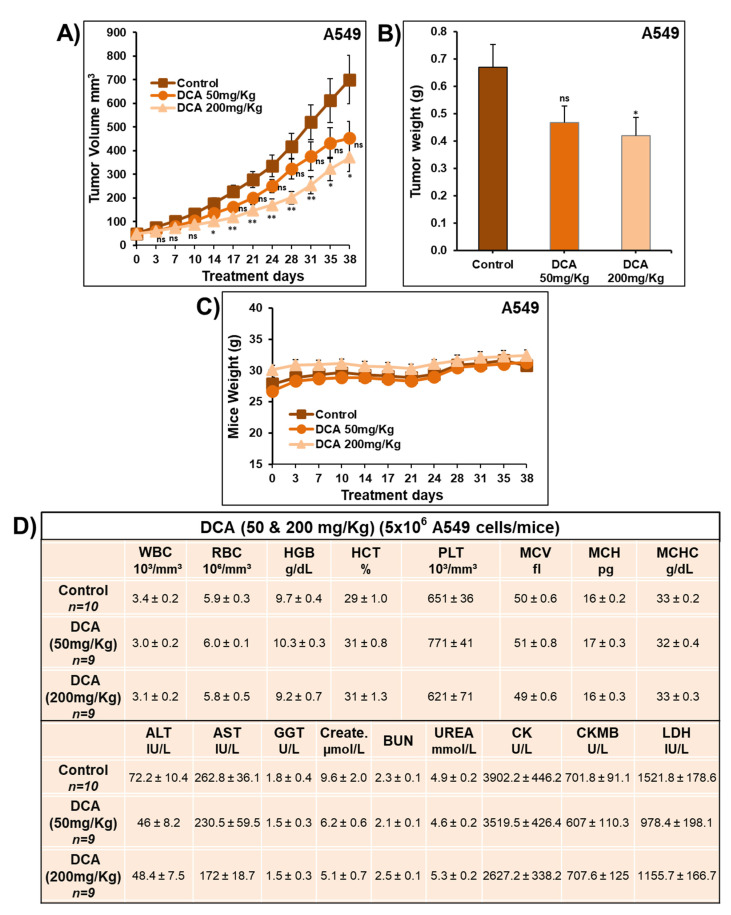
Effect of DCA on the growth of A549 xenografted in nude mice in vivo. (**A**) Tumor volume of A549 xenograft inoculated subcutaneously in nude mice and treated with DCA (50 and 200 mg/kg) orally or control carrier solution alone for a total of 38 days. (**B**) Tumor weight obtained from the same control and DCA-treated nude mice. (**C**) Average of the mice body weight through the treatment days. (**D**) Mice blood samples were analyzed for complete blood count, liver and kidney function parameters. Results represent mean ± S.E.M. of 9–10 mice/group. * Significantly different at <0.05. ** Significantly different at <0.01. ns—non-significant.

The impact of DCA on tumor xenografts was also evaluated in vivo using athymic mice inoculated with A549 and LNM35 cells. The median lethal doses (LD50) of DCA were reported to be 4.5 g/kg and 5.5 g/kg in rats and mice, respectively [17]. Therefore, mice with A549 tumor xenografts were treated orally everyday (5 days/week) with 50 mg/kg and 200 mg/kg of DCA for 38 consecutive days. Treatment with DCA (50 mg/kg) did not cause a significant reduction in the volume of A549 tumor xenografts, while DCA (200 mg/kg) significantly reduced the volume by approximately 45% (Figure 3A). A similar difference was also observed in tumor weight at the end of the experiment (Figure 3B). There were no obvious signs of toxicity or any manifestation of undesirable effects of DCA on animal behavior, body weight (Figure 3C), blood components, liver and kidney function (Figure 3D).

**Figure 4:**
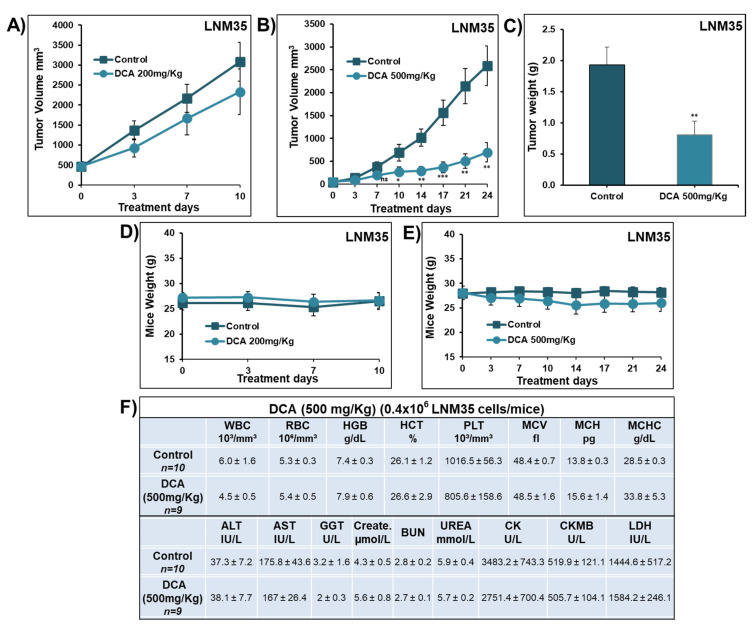
Effect of DCA on the growth of LNM35 xenografted in nude mice in vivo. (**A**,**B**) Tumor volume of LNM35 xenograft inoculated subcutaneously in nude mice and treated, respectively, with DCA (200 and 500 mg/kg) orally or control carrier solution alone daily for a total of 10 and 24 days. (**C**) Tumor weight obtained from the control and 500 mg/kg DCA-treated nude mice. (**D**,**E**) Average of the mice body weight throughout the treatment days. (**F**) Mice blood samples were analyzed for complete blood count, liver and kidney function parameters. Results represent mean ± S.E.M. of 9–11 mice/group. * Significantly different at <0.05. ** Significantly different at <0.01. *** Significantly different at <0.001. ns—nonsignificant.

On the other hand, the growth of LNM35 tumor xenografts was monitored, and the mice were treated orally with 200 mg/kg and 500 mg/kg of DCA every day (5 days/week) for 10 and 24 days, respectively. Treatment with DCA (200 mg/kg) caused a non-significant reduction in the volume of LNM35 tumor xenografts (Figure 4A), while DCA (500 mg/kg) significantly decreased the tumor volume by nearly 75% (Figure 4B). Almost similar differences were seen in the tumor weight at the end of the experiments (Figure 4C). No signs of toxicity were observed in the animals’ behaviors or detected from the mice’s weight (Figure 4D,E), blood components, liver, and kidney function (Figure 4F). 

### 2.3. Effect of DCA on the Formation of Capillary-Like Structures and Sprouting by HUVECs In Vitro

**Figure 5:**
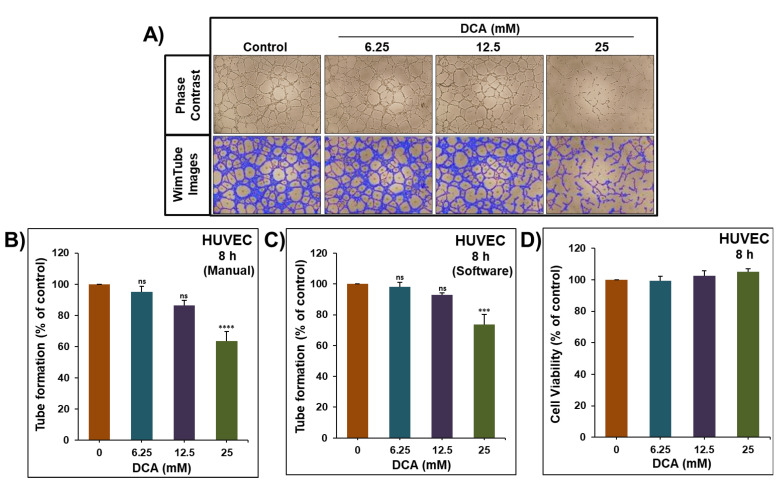
Effect of DCA on the formation of capillary-like structures by HUVECs in vitro. (**A**) Forms of angiogenesis induced in HUVEC cultured on Matrigel matrix in 96-well plate in the absence and presence of different concentrations of DCA. An inverted microscope (4×) was used for contrast photo and Wimasis software was used to clarify the pictures. (**B**,**C**) Quantification of tubular angiogenesis induced in HUVEC cells cultured in the absence and presence of DCA (6.25–25 mM) manually and by using Wimasis software, respectively. (**D**) HUVEC cells’ viability was determined as described in the Material and Methods in the absence and presence of DCA (6.25–25 mM). Experiments were repeated at least 3 times. Columns represent means; bars represent S.E.M. *** Significantly different at <0.001. **** Significantly different at <0.0001. ns—non-significant.

Angiogenesis is one of the cancer hallmarks that ensures the supply of nutrients and oxygen for the cancer cells to grow and spread. The impact of DCA on angiogenesis was investigated in vitro using HUVECs that can form capillary-like structures when seeded on Matrigel. As shown in Figure 5A, HUVECs formed organized capillary-like structures in the absence of DCA, and this organization was disturbed after DCA addition. Tubes’ lengths were measured manually (Figure 5B) and by using Wimasis Image Analysis (Figure 5C), and it was found that 25 mM of DCA was able to significantly inhibit the HUVECs’ capacity to form the threaded structures by nearly 30–40%. This inhibition was observed with concentrations that did not show any reduction in HUVECs’ viability (Figure 5D).

**Figure 6:**
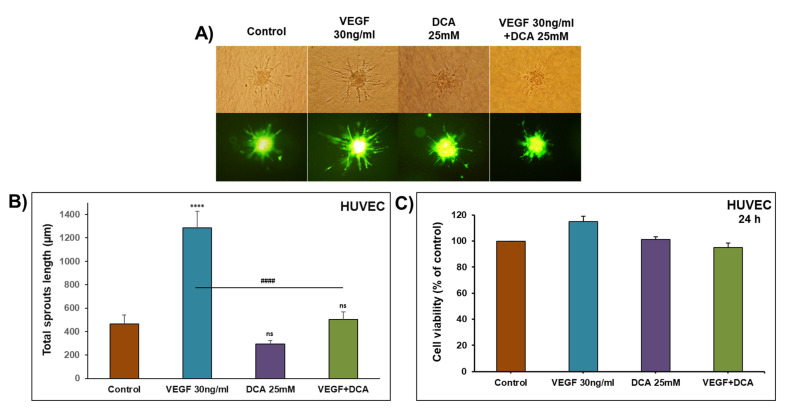
Effect of DCA on the formation of sprouts by the embedded HUVECs spheroids in vitro. (**A**) Representative images of pre-dyed HUVEC spheroids after 24 h of embedding in collagen matrix in the presence of VEGF 30 ng/mL, DCA 25 mM or VEGF + DCA. An inverted microscope at 20× magnification was used. (**B**) Average of total sprout lengths from different spheroids per condition from one representative experiment. (**C**) HUVECs’ viability was determined as described in the Material and Methods. Experiments were repeated 2 times. Columns represent means of 12 spheroids; bars represent S.E.M. **** Significantly different at <0.0001. #### Significantly different at <0.0001. ns: non-significant.

In the sprouting assay, spheroids of HUVECs were embedded in a 3D collagen matrix in the presence and absence of VEGF 30 ng/mL, DCA 25 mM or a combination of VEGF and DCA. Figure 6A shows in a representative experiment that the sprouts formed in the presence of VEGF were inhibited by DCA, 25 mM. Total sprout lengths were measured, and it was found that the total length was significantly increased in the presence of VEGF, and DCA significantly decreased the sprout lengths induced by VEGF (Figure 6B). This inhibition was observed with a concentration that did not show any reduction in HUVECs’ viability (Figure 6C).

These data suggest that the inhibition of tumor angiogenesis could be a potential mechanism beyond the anti-cancer effects of DCA.

### 2.4. Effect of DCA on NSCLC Metastasis In Vivo and Invasion and Migration In Vitro

**Figure 7:**
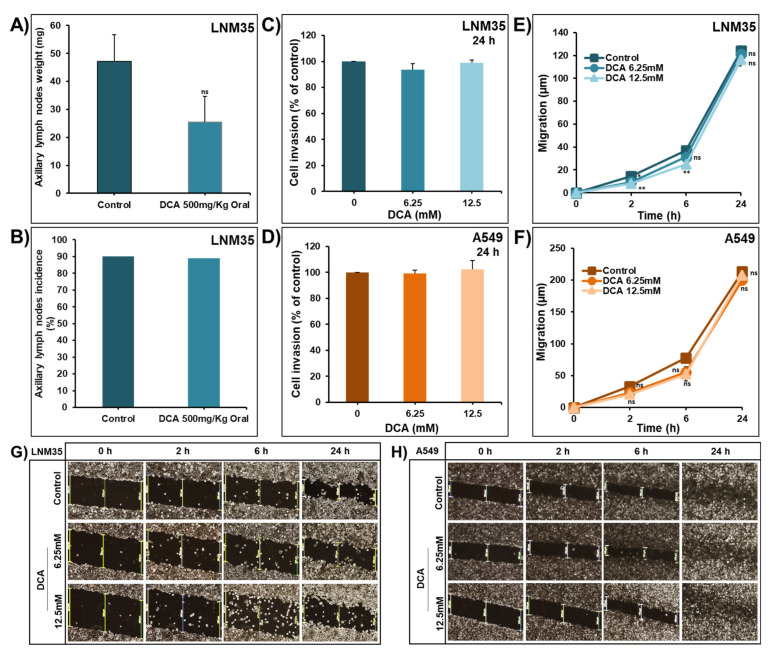
Effect of DCA on NSCLC metastasis in vivo and invasion and migration in vitro. (**A**) Weight of the axillary lymph nodes with LNM35 metastases in control and DCA-treated groups (500 mg/kg oral). (**B**) Percentage of mice with LNM35 lymph node metastases in control and DCA-treated groups. Results represent mean ± SEM of 9–10 mice/group. Using Boyden invasion chamber assay, LNM35 (**C**) and A549 (**D**) cells were incubated for 24 h in the absence and presence of DCA (6.25, 12.5 mM). Cells that invaded into the Matrigel and crossed the 8 µm pores were determined as described in the Materials and Methods. Scratches were introduced in confluent monolayers of LNM35 cells (**E**) and A549 cells (**F**) cultured in 6-well plate in the absence and presence of DCA (6.25, 12.5 mM). An inverted microscope with 4× magnification was used to measure the average distance that cells migrated from the edge of the scrapped area for 2, 6, and 24 h. Pictures of induced scratches in the confluent monolayers of LNM35 cells (**G**) and A549 cells (**H**) in the presence and absence of different concentrations of DCA at 0, 2, 6 and 24 h. All experiments were repeated at least 3 times. Columns or shapes are means; bars are S.E.M. * Significantly different at <0.05. ** Significantly different at <0.01. ns—non-significant.

Metastasis is a multistep process comprised of cell detachment from the primary tumor, cells migration to the adjacent tissues followed by cells invasion into the blood or lymphatic system until the colonization of these cells in the distant organs. The effect of DCA on metastasis in mice xenografted with the highly metastatic lung cancer cells, namely, LNM35, was evaluated by checking the weight and incidence of axillary lymph nodes in the control and DCA-treated groups. DCA decreases the growth of lymph node metastases without reaching a statistical significance (Figure 7A). In addition, it did not affect the incidence of lymph node metastases (Figure 7B).

A Boyden chamber invasion assay and wound-healing migration assay were used to evaluate the ability of DCA to inhibit A549 and LNM35 cell invasion and migration in vitro. To ensure that the potential effect of DCA on migration and invasion is not due to cell death, we used lower concentrations of DCA. Under these conditions, 6.25 mM and 12.5 mM of DCA failed to inhibit the cellular invasion of LNM35 (Figure 7C) and A549 (Figure 7D). Similarly, these concentrations were unable to inhibit the cellular migration of both cell lines (Figure 7E–H).

### 2.5. Effect of DCA in Combination with Chemotherapeutic Agents on the Viability of NSCLC Cells

**Figure 8:**
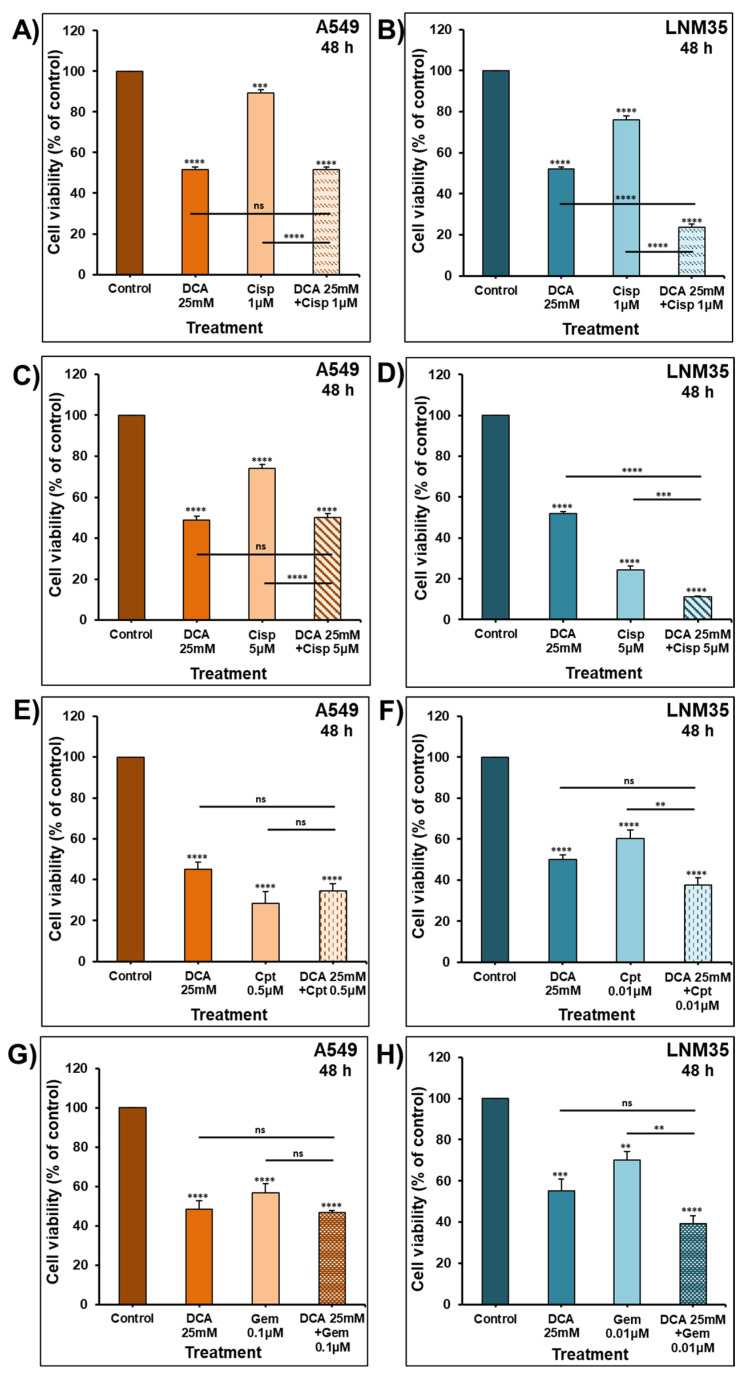
Effect of DCA in combination with chemotherapeutic agents on the viability of NSCLC cells. Exponentially growing A549 cells (**A**,**C**) and LNM35 cells (**B**,**D**) were treated, in 96-well plate for 48 h, with DCA (25 mM) ± cisplatin (1, 5 µM). Similarly, exponentially growing A549 cells were treated, in 96-well plate for 48 h, with DCA (25 mM) in combination with camptothecin (0.5 µM) (**E**) or gemcitabine (0.1 µM) (**G**) while LNM35 cells were treated with camptothecin (0.01 µM) (**F**) or gemcitabine (0.01 µM) (**H**). Cellular viability was determined using CellTiter Glo luminescent assay as described in the Material and Methods. Experiments were repeated at least 3 times. Columns represent means; bars represent S.E.M. ** Significantly different at <0.01. *** Significantly different at <0.001. **** Significantly different at <0.0001. ns—non-significant.

To further evaluate the therapeutic potential of DCA, we investigated whether its anti-cancer effects could enhance the anti-cancer activity of major chemotherapeutic drugs, namely, cisplatin, camptothecin and gemcitabine. The treatment of the cells for 48 h with 25 mM of DCA failed to enhance the anti-cancer effects of cisplatin (1 µM) in A549 cancer cells (Figure 8A). In contrast, it significantly enhanced the inhibitory effect of the same concentration of cisplatin in LNM35 cancer cells (Figure 8B). Similar results were also obtained when used in combination with a higher concentration of cisplatin (5 µM) (Figure 8C,D). Additionally, 25 mM of DCA did not enhances the anti-cancer effects of camptothecin (0.5 µM and 0.01 µM) and gemcitabine (0.1 µM and 0.01 µM) in both cell lines (Figure 8E–H).

### 2.6. Effect of DCA in Combination with EGFR-TKi on NSCLC Cellular Viability and Colony Growth

**Figure 9:**
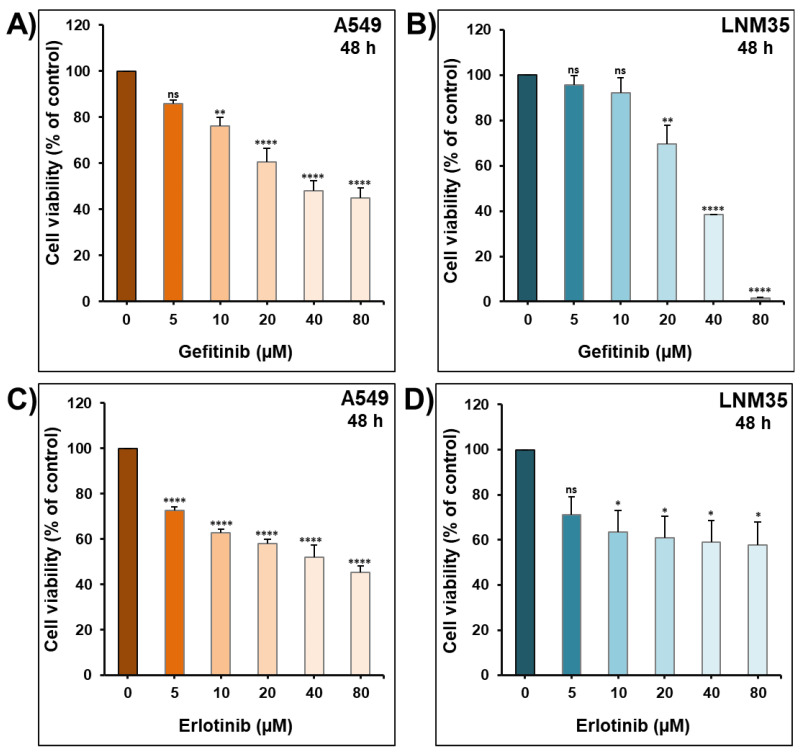
Effect of EGFR-Tki on NSCLC cells’ viability. Exponentially growing A549 (**A**,**C**) and LNM35 (**B**,**D**) cells were treated with drug vehicle, gefitinib or erlotinib (5–80 µM) for 48 h. Cellular viability was determined using CellTiter-Glo luminescent assay as described in the Materials and Methods. Experiments were repeated at least 3 times. Columns are means; bars are S.E.M. * Significantly different at <0.05. ** Significantly different at <0.01. **** Significantly different at <0.0001. ns—non-significant.

The impact of 48 h incubation with increasing concentrations of gefitinib and erlotinib (5–80 µM) was investigated on A549 and LNM35 cancer cells. Gefitinib caused a concentration-dependent reduction in the viability of A549 and LNM35 cancer cells (Figure 9A,B); likewise, erlotinib showed the same reduction pattern in the two cell lines (Figure 9C,D). An amount of 20 µM of gefitinib and erlotinib has the ability in both cell lines to inhibit the cellular viability of A549 and LNM35 by approximately 40%, and this concentration was used in the combination experiments with DCA.

**Figure 10:**
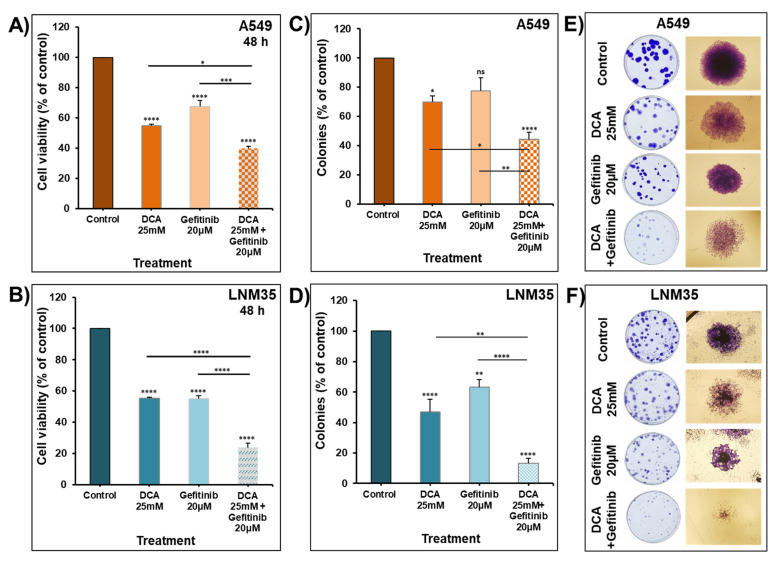
Effect of DCA in combination with gefitinib on NSCLC cells’ viability and colony growth. Exponentially growing A549 (**A**) and LNM35 (**B**) cells were treated, respectively, with DCA (25 mM) ± gefitinib 20 µM. Cellular viability was determined using CellTiter-Glo luminescent assay. (**C**,**D**) Treatment of the pre-formed colonies of A549 and LNM35 cells, respectively, with DCA (25 mM) ± gefitinib 20 µM for 7 days, after which colonies were fixed, stained and counted as described in the Materials and Methods. (**E**,**F**) Representative images of the colonies for the control and treated groups are shown for A549 and LNM35 cancer cells. All experiments were repeated at least 3 times. Columns are means; bars are S.E.M. * Significantly different at <0.05. ** Significantly different at <0.01. *** Significantly different at <0.001. **** Significantly different at <0.0001. ns—non-significant.

The treatment of the cells for 48 h with 25 mM of DCA significantly enhanced the effect of gefitinib on the cellular viability of A549 (Figure 10A) and LNM35 (Figure 10B). Next, a clonogenic assay was conducted to evaluate the effect of the combination on the growth of pre-formed colonies of both cell lines after seven days of treatment. The concentration of 20 µM of gefitinib caused a 20–40% reduction in the number of A549 (Figure 10C) and LNM35 (Figure 10D) colonies. Compared to the individual treatments, the combination of DCA with gefitinib leads to a significant reduction in the number of colonies of both cell lines (Figure 10C,D), causing an additive effect in LNM35 when compared to the calculated additive value of single treatments (86% vs. 90%). In addition, this combination shows a significant decrease in the cell density of the individual colonies of both cell lines (Figure 10E,F). 

**Figure 11:**
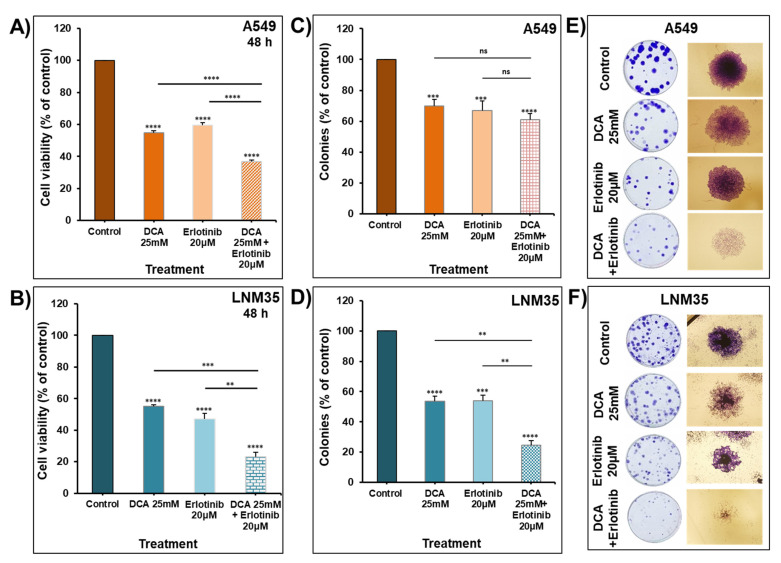
Effect of DCA in combination with erlotinib on NSCLC cells’ viability and colony growth. Exponentially growing A549 (**A**) and LNM35 (**B**) cells were treated, respectively, with DCA (25 mM) ± erlotinib 20 µM. Cellular viability was determined using CellTiter-Glo luminescent assay. (**C**,**D**) Treatment of the pre-formed colonies of A549 and LNM35 cells, respectively, with DCA (25 mM) ± erlotinib 20 µM for 7 days, after which colonies were fixed, stained and counted as described in the Materials and Methods. (**E**,**F**) Representative images of the colonies for the control and treated groups are shown for A549 and LNM35 cancer cells. All experiments were repeated at least 3 times. Columns are means; bars are S.E.M. ** Significantly different at <0.01. *** Significantly different at <0.001. **** Significantly different at <0.0001. ns—non-significant.

Similarly, DCA enhances the inhibitory effect of erlotinib on the cellular viability of A549 and LNM35 (Figure 11A,B). The number of A549 and LNM35 colonies was significantly reduced with erlotinib by 30–40% (Figure 11C,D), and this reduction was enhanced by DCA in LNM35 (Figure 11D) but not A549 (Figure 11C). The combination caused additive effects in LNM35 by decreasing the number of colonies by 76 ± 2.8%, which is statistically non-significant from the calculated additive value of single treatments (91 ± 5.8%). Despite the non-significant reduction in the number of A549 colonies with the combination compared to single-drug treatments, the cell density of each colony was significantly reduced compared to the individual treatments (Figure 11E). Likewise, the cell density of the LNM35 colonies was reduced in the combination-treated group (Figure 11F).

**Figure 12:**
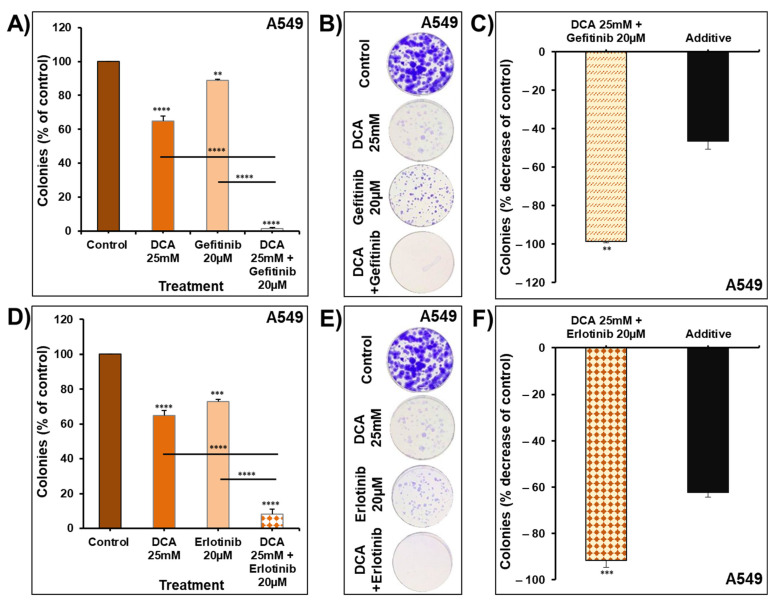
Effect of longer duration treatment with DCA in combination with gefitinib and erlotinib on A549 colony growth. Treatment of the pre-formed colonies of A549 with DCA (25 mM) ± gefitinib 20 µM (**A**,**B**) and DCA (25 mM) ± erlotinib 20 µM (**D**,**E**) for 14 days, after which colonies were fixed, stained and counted as described in the Materials and Methods. (**C**,**F**) Effect of combinations of DCA and gefitinib or erlotinib on colony growth compared with the calculated additive effects of the two drugs alone. All experiments were repeated 3 times. Columns are means; bars are S.E.M. ** Significantly different at <0.01. *** Significantly different at <0.001. **** Significantly different at <0.0001.

To address the differences between the two cell lines in the impact of the combination therapy on the colony growth, a longer duration of treatment with DCA in combination with gefitinib or erlotinib on A549 colony growth was investigated. As shown in Figure 12, the combination of DCA with gefitinib leads to a significant reduction in the number of colonies (Figure 12A,B). This combination produced a greater inhibition in the number of colonies compared to the calculated additive effects of the drugs used alone (Figure 12C). A similar observation was noticed with the combination of DCA and erlotinib (Figure 12D–F). In conclusion, increasing the duration of the treatments from seven to fourteen days leads to synergistic effects of the suggested combination protocols. 

## 3. Discussion

Despite the recent advances in the screening, diagnosis and management of lung cancer, in addition to the remarkable progress in understanding its molecular biology, lung cancer is the second most commonly diagnosed cancer with the highest mortality rate worldwide in 2020 [1]. Therefore, various efforts are being devoted to the development of effective agents and approaches with good safety margins to target lung cancer in an attempt to provide a cure or improve the patient’s overall survival. This study aimed to investigate the impact of the metabolic drug DCA on lung cancer growth, migration, invasion and angiogenesis in vitro and tumor growth and metastasis in vivo as well as the effect of targeting metabolism by DCA on the cytotoxic effect of approved chemotherapy and targeted therapy as a step to achieve a better efficacy and better safety profile.

The present study showed that DCA (3.125–100 mM) produced a concentration- and time-dependent reduction in the cellular viability and growth of pre-formed colonies of A549 and LNM35 cell lines. The IC50 of DCA at 48 h was approximately 25 mM in both cell lines. Our results are in agreement with other reports in which DCA (10–90 mM) inhibited the cellular viability of colorectal cancer (CRC) cell lines, namely, SW620, LS174t, LoVo and HT-29 in a concentration-dependent manner at 48 h with an IC50 range of 30–50 mM according to the cell line type [18]. Similarly, DCA (20 mM) significantly decreased the viability of CRC cells, namely, SW480, LoVo and HT-29 at 48 h, with a greater effect on the poorly differentiated SW480 cells and metastatic LoVo cells compared to the well-differentiated HT-29 cells [19]. On the other hand, a higher IC50 was reported in cervical cancer cells, Hela and SiHa cells [20], while DCA (20 mM) failed to inhibit the cellular viability of the breast cancer MCF-7 cell line [21].

Our in vitro data were validated by testing the effect of DCA on tumor progression in vivo using a chick embryo CAM and athymic mice models. Firstly, we demonstrated that a significant growth reduction was achieved in the A549 and LNM35 xenografted on chick embryo CAM by using DCA doses of 50 mM and 100 mM, respectively. During the writing of this manuscript, a study was published that investigated the effect of sodium DCA on U87 MG and PBT24 glioblastoma cell lines xenografted on a chick embryo CAM [22]. The authors reported a variation in U87 MG and PBT24 tumor growth in response to the different concentrations of sodium DCA. It was reported that 10 mM of sodium DCA was effective in reducing the PBT24 tumor growth but not U87 tumor growth, reflecting some differences in the biology of the two cell lines [22]. Secondly, we demonstrated that treatment with DCA at doses of 200 mg/kg everyday (5 days/week) caused a significant 40% reduction in xenografted A549 tumor growth, while a higher dose of DCA (500 mg/kg) was required to produce a significant decrease in xenografted LNM35 tumor growth. In this context, it has been previously reported that DCA (100 mg/kg) increased the tumor doubling time of A549 and H1975 NSCLC from approximately 3 to 6.5 days [15], but failed to produce a significant inhibitory effect in MDA-MB-231 tumor-bearing mice [23]. On the other hand, a significant growth delay was also observed in HT-29 xenografts treated with oral DCA (200 mg/kg) daily for four days [24]. 

Investigating the toxicity of the potential anti-cancer drugs is as important as investigating their efficacy since severe toxicity can prevent their use in the clinic. DCA showed no cytotoxicity to the chick embryos and athymic mice. The percentage of alive embryos was the same in the DCA-treated and control groups. Additionally, DCA did not affect mice behavior, weight, complete blood count, liver and kidney function parameters compared to the control group. These findings are consistent with previous preclinical and clinical reports that showed no evidence of severe hematologic, hepatic, renal, or cardiac toxicity with DCA treatment [13,14]. Few patients treated with DCA complained of common gastrointestinal effects. Additionally, the most common limitation to DCA administration is reversible peripheral neuropathy, which can be minimized by dose reduction or the complementary administration of antioxidants [11]. Incorporating DCA into drug delivery systems (DDS) such as nanoparticles is a promising approach to retain the anti-cancer activity of DCA with minimal side effects [25,26,27]. 

The anti-cancer effect of DCA was reported to be partly due to the induction of apoptosis, as was observed in colorectal cancer cells [19] and NSCLC cells [15] or due to the inhibition of angiogenesis. Angiogenesis inhibitors such as the anti-VEGF antibody Bevacizumab and VEGF receptor blocker Ramucirumab have been approved clinically for the management of lung cancer [28]. Despite their approved efficacy, their modest overall therapeutical effects with the associated side effects highlight a clear need for a more effective approach targeting angiogenesis [28]. Our study demonstrated that DCA (25 mM) is a promising anti-angiogenic agent by being able to significantly inhibit endothelial cell tube formation and sprouting in vitro. In addition, lower concentrations of DCA (6.25 and 12.5 mM) did not affect the HUVECs tube formation. These findings are consistent with a report by Schoonjans and coworkers, who demonstrated that 5 mM and 10 mM DCA did not affect HUVECs’ tube formation in vitro [29]. In agreement with our data, DCA caused a reduction in the tumor microvessel density in treated rats, in which HIF1α suppression was also reported within the tumor cells [30]. On the other hand, Zhao and coworkers recently reported that DCA stimulates angiogenesis in a vascular dementia rat model by improving endothelial precursor cell function [31].

Approximately 30–40% of NSCLC patients presented with metastatic disease at the time of diagnosis. Distant metastases negatively affect the treatment options, response and survival [32] and are the main cause of lung cancer deaths [33]. Metastasis is a multistep process involving the detachment of cancer cells, migration, invasion and colonization at distant sites. Therefore, therapeutic agents and regimens reducing such a hallmark in cancer are of high importance in cancer therapy. Despite the demonstrated anti-angiogenic activity of DCA, this study showed no impact of DCA on the metastasis of LNM35 cells xenografted in athymic mice treated orally with an effective dose. In this study, LNM35 cells xenografted by subcutaneous inoculation in athymic mice caused a 90% incidence of axillary lymph node metastases, and DCA failed to reduce the incidence and the growth of these lymph node metastases. The LNM35 cell line was established in 2000 as the first human lung cancer cell line having lymphogenous metastatic properties with 100% incidence following a subcutaneous injection into the lateral flank of nude mice [34]. Additionally, DCA did not show any inhibitory effects on the migratory and invasive properties of LNM35 and A549 cells in vitro. Similarly, it was reported that DCA monotherapy was not effective in reducing lung metastases from metastatic breast cancer cells xenografted in nude mice [23]. 

Combination therapy has been a fundamental approach in cancer management. Combining different anti-cancer drugs allows the targeting of different essential signaling pathways to enhance therapeutic benefits, avoid the acquired resistance and decrease the severity of side effects [35]. Chemotherapy plays an integral part in the management of NSCLC patients. A regimen of platinum (cisplatin or carboplatin) plus paclitaxel, gemcitabine, docetaxel, vinorelbine, irinotecan, or pemetrexed is usually used [36]. The nonselective characteristics of chemotherapeutic agents results in a modest increase in survival with significant toxicity to the patient [37]. This underscores the need for better strategies to improve patients’ outcomes with minimal side effects. In the present study, DCA failed to enhance the anti-cancer effect of camptothecin and gemcitabine in both NSCLC cell lines. Additionally, DCA failed to significantly enhance the anti-cancer effects of cisplatin in the A549 cell line in vitro, but it enhanced the cytotoxic effect of cisplatin in the LNM35 cell line, reflecting the role of the genetic background of cancer cells in determining the cell death pathway induced by the drugs. Kim et al. reported that A549 cells have a lower rate of aerobic glycolysis compared to H460 cells due to differential expression in some metabolic enzymes [38]. Aerobic glycolysis in cancer has been linked to chemoresistance, and the inhibition of related pathways has been suggested as a mechanism for overcoming such resistance. For instance, the overexpression of PDK4 in high-grade bladder cancer makes the co-administration of DCA with cisplatin cause a dramatic reduction in tumor growth compared to DCA or cisplatin alone [39]. Similarly, the administration of DCA with paclitaxel was reported as a successful approach to overcome the paclitaxel-resistant NSCLC cells due to PDK2 overexpression [40]. Furthermore, Galgamuwa et al. stated that pre-treatment with DCA significantly attenuated the nephrotoxicity induced by cisplatin in mice, retaining the cisplatin anti-cancer effects [41]. 

The discovery of targeted therapy has helped physicians to tailor the treatment options for NSCLC patients. Many targeted drugs have been developed and become part of the first-line treatment of NSCLC, such as gefitinib and erlotinib, which are considered the first generation of EGFR-TKi [42]. Gefitinib and erlotinib were approved more than 10 years ago for the treatment of chemotherapy-naive patients with advanced EGFR-mutant NSCLC as the first-line treatment. They are also used as a second-line therapy after chemotherapy failure [43]. Some reports showed that erlotinib has good efficacy in patients with EGFR-wild-type NSCLC [44]. A maintenance dose can benefit these patients after platinum-based chemotherapy, considered the backbone therapy in wild-type EGFR NSCLC [45]. Despite the remarkable benefits, many patients acquired therapeutic resistance after 10–14 months of treatment due to a secondary mutation in the EGFR gene [46]. 

In this study, we were seeking to investigate the ability of DCA to sensitize the EGFR wild-type NSCLC cell lines when combined with gefitinib or erlotinib in vitro. DCA significantly enhanced the inhibitory effect of gefitinib and erlotinib on the cellular viability of A549 and LNM35. This study also showed additive effects on LNM35 colony growth upon combining DCA with gefitinib or erlotinib for seven days of treatment. Furthermore, this combination produced synergistic effects on A549 colony growth after fourteen days of treatment. In addition, all these combination protocols lead to a substantial decrease in the cellular density of individual colonies of both A549 and LNM35. In this context, it has been reported that DCA with gefitinib or erlotinib synergistically inhibits the viability and colony formation capacity of EGFR-mutant cells (NCI-H1975 and NCI-H1650) due to synergistic effect in promoting apoptosis. In EGFR wild-type cells (A549 and NCI-H460), they showed, in comparison to the individual treatments, that combination caused an elevated fraction affected (Fa) value in cellular viability without reaching the level of synergism in EGFR wild-type cells (A549 and NCI-H460), and this combination did not significantly repress the colony formation of these cell lines [47]. The differences in the experimental conditions between the aforementioned report and our study could explain such variable results. In their clonogenic assay, the investigators treated the individual cells for three successive days, followed by incubation with a drug-free medium for 15 days to form colonies; however, in our experiments, the cells were firstly incubated for ten days to form colonies followed by seven and fourteen days of treatment. 

In summary, this study demonstrated that DCA is a promising anti-cancer agent for NSCLC by inhibiting the cellular viability and colony growth of NSCLC cells in vitro and tumor growth in the chick embryo CAM and nude mice, in which the safety of this agent was also assessed. DCA inhibits the ability of endothelial cells to form capillary-like structures and sprouting in vitro, suggesting the inhibition of angiogenesis as a potential mechanism behind the anti-cancer effect. This study also revealed the potential value of DCA when combined with gefitinib or erlotinib in vitro. The findings of this study pave the way for validating the impact of the combination of DCA with gefitinib or erlotinib on tumor growth in vivo, in addition to investigating the impact of DCA when combined with the second- and third-generation EGFR-TKi.

## 4. Materials and Methods

### 4.1. Cell Culture and Reagents

NSCLC cells, A549 and LNM35, were maintained in RPMI-1640 medium (Gibco, Paisley, UK) in a humidified incubator at 37 °C and 5% CO_2_. The medium was supplemented with 1% of penicillin–streptomycin solution (Hyclone, Cramlington, UK) and 10% of fetal bovine serum (Hyclone, Cramlington, UK). Human umbilical vein endothelial cells (HUVECs) were maintained in an EndoGRO^TM^-VEGF complete media kit (Merck Millipore, Massachusetts, USA) in a humidified incubator at 37 °C and 5% CO_2_ in flasks coated with 0.2% gelatin. The culture medium of all cells was changed every 3 days, and cells were passed once a week when the culture reached 95% confluency for cancer cells and 80% for HUVECs.

Sodium DCA, cisplatin, camptothecin, gemcitabine HCl, erlotinib HCl and gefitinib were purchased from Sigma-Aldrich (Saint Louis, MO, USA). DCA was freshly dissolved in HyPure water (Hyclone, Cramlington, UK) before starting any experiment to make a stock solution of 1M, which was then diluted to the required concentrations for treatment.

### 4.2. Cellular Viability

A549 and LNM35 cells were seeded at a density of 5000 cells/well into a 96-well plate. After 24 h, cells were treated with an increasing concentration of DCA (3.125–100 mM) in duplicate for 24, 48 and 72 h, whereas control cells were treated with a drug vehicle (Hypure water) mixed with medium. At the indicated time points, a CellTiter-Glo^®^ Luminescent Cell Viability assay (Promega Corporation, Madison, WI, USA) was used to determine the effect of DCA on cellular viability by quantifying the ATP that will be proportional to the number of the metabolically active cells. The luminescent signal was measured by a GloMax^®^ Luminometer (Promega Corporation, Madison, WI, USA). Cellular viability was presented as a percentage (%) by comparing the viability of DCA-treated cells to the control cells, the viability of which is assumed to be 100%.

In the second set of experiments, cells were treated for 48 h with an increasing concentration of gefitinib and erlotinib (5–80 µM). Additionally, cells were treated for 48 h with a combination of DCA and other anti-cancer agents, namely, cisplatin, camptothecin, gemcitabine, gefitinib and erlotinib. Cellular viability was determined using a CellTiter-Glo^®^ Luminescent Cell Viability assay and the GloMax^®^ Luminometer (Promega Corporation, Madison, WI, USA). The viability was presented as a percentage (%) by comparing the viability of drug-treated cells with the control cells.

### 4.3. Clonogenic Assay 

Into a 6-well plate, A549 and LNM35 cells were seeded, respectively, at 50 and 100 cells/well. Cells were kept to grow into colonies for 7–10 days in a humidified atmosphere at 37 °C and 5% CO_2_, with the medium being changed every three days. Formed colonies were treated every 3 days for 7 days with increasing concentrations of DCA (6.25–50 mM). Afterwards, colonies were washed three times with 1× PBS, fixed and stained for 2 h with 0.5% crystal violet dissolved in 50% methanol (*v*/*v*). Finally, colonies were washed with 1× PBS and photographed, and colonies with more than 50 cells were counted. Data are presented as the colony percentage (%) by comparing the DCA-treated colonies with the control colonies. Colony cell density was assessed by photographing the colonies in each group using an inverted phase-contrast microscope (4×).

In the second set of experiments, formed colonies were treated every 3 days for 7 or 14 days with a combination of DCA and gefitinib or DCA and erlotinib. Data are presented as colonies percentage (%) by comparing the drug-treated colonies with the control colonies.

### 4.4. In Ovo Tumor Growth Assay

Fertilized Leghorn eggs were incubated in the egg incubator set at a temperature of 37.5 °C and humidity of 50% for the first 3 days after fertilization. At embryonic day 3 (E3), the CAM was dropped by drilling a small hole into the eggshell opposite to the round, wide end followed by aspirating ~1.5–2 mL of albumin using a 5 mL syringe with 18G needle. Then, a small window was cut into the eggshell above the CAM using a delicate scissor and sealed with a semipermeable adhesive film (Suprasorb^®^ F). The eggs were kept again in the egg incubator until embryonic day 9 (E9), in which cancer cells were trypsinized, centrifuged and suspended in an 80% Matrigel^®^ Matrix (Corning, Bedford, UK) to have 1 × 10^6^ cells/100 µL for A549 and 0.3 × 10^6^ cells/100 µL for LNM35. A 100 µL inoculum was added onto the CAM of each egg for a total of 10–13 eggs per condition. At embryonic day 11 (E11), formed tumors were treated topically by dropping 100 µL of the DCA prepared in 0.9% NaCl for the first group or the drug vehicle for the control group. Treatment was repeated at E13 and E15. All the described steps were performed under aseptic conditions. Finally, at embryonic day 17 (E17), embryos were humanely euthanized by a topical addition of 10–30 µL pentobarbitone sodium (300 mg/mL, Jurox, Auckland, New Zealand). Tumors were carefully extracted from the normal upper CAM tissues, washed with 1× PBS and weighted to determine the effect of DCA on tumor growth. Data are presented as comparisons of the average weight of tumors in the control group and DCA-treated group. Drug toxicity was assessed by comparing the percentage of alive embryos in the control and DCA-treated groups at the end of the experiment. Alive embryos were determined by checking the voluntary movements of the embryos in addition to the integrity and pulsation of the blood vessels. 

This assay is a randomly assigned unblinded assay that was carried out according to the protocol approved by the animal ethics committee at the United Arab Emirates University. According to the European Directive 2010/63/EU on the protection of animals used for scientific purposes, experiments involved using chicken embryos on and before E18, do not require approvals from the Institutional Animal Care and Use Committee (IACUC). 

### 4.5. Tumor Growth and Metastasis Assay

The animal experiments were performed according to the protocol approved by the UAE university animal ethics committee in March 2019 (protocol code ERA_2019_5872). Six- to eight-week-old athymic NMRI male nude mice (nu/nu, Charles River, Germany) were housed in filtered-air laminar flow cabinets and handled under aseptic conditions. A549 cells (5 × 10^6^ cells/200 µL PBS) and LNM35 cells (0.4 × 10^6^ cells/200 µL PBS) were injected subcutaneously into the lateral flank of the nude mice. Ten days later, when tumors had reached the volume of approximately 50 mm^3^, animals with A549 xenografts were divided randomly into three groups of 9–10 mice each. These groups were treated orally every day (5 days/week) with DCA 50 mg/kg or 200 mg/kg or drug vehicle for 38 days. On the other hand, animals with LNM35 xenografts were treated orally every day (5 day/week) with DCA 200 mg/kg or drug vehicle for 10 days and DCA 500 mg/kg or drug vehicle for 24 days. Tumor dimensions and animal weights were checked every three or four days. In addition, the physical signs and behavior were checked every day. Tumor volume was calculated using the formula V = L × W^2^ × 0.5, with L representing the length and W the width of the tumor. At the end of the experiments, animals were anesthetized and sacrificed by cervical dislocation, and tumors were removed and weighted. The effect of DCA on tumor growth was presented by comparing the average tumor weight at the end of the experiment between the control group and the DCA-treated group. It was also assessed by comparing the tumor volume between the control and DCA-treated groups throughout the experiment. Blood samples were collected from each mouse and analyzed using the SCIL VET ABC™ Animal Blood Counter for a complete blood count. In addition, blood plasma was separated by centrifugation for biochemical analysis. To study the impact of DCA on metastasis, axillary lymph nodes were excised and weighted from the animals with LNM35 xenografts at the end of the experiment.

### 4.6. Vascular Tube Formation Assay

Matrigel^®^ Matrix (Corning, Bedford, UK) was thawed, and 40–50 µL was added to the wells of a 96-well plate for coating. In order for the Matrigel to solidify, the plate was kept in a humidified incubator at 37 °C and 5% CO_2_ for 1 h. HUVECs were trypsinized and seeded on the coated plate at a density of 2.5 × 10^4^ cells/100 µL/well in the presence and absence of different concentrations of DCA. After 8 h of incubation, the tube networks at the different wells were photographed using an inverted phase-contrast microscope. The impact of DCA on the ability of HUVECs to form capillary-like structures was assessed by measuring the total lengths of the formed tubes in the control and DCA-treated wells. Total tube lengths were measured manually and through an online image analysis software developed by Wimasis (https://www.wimasis.com/en/products/13/WimTube - access date 1 March 2019). The effect of the different concentrations of DCA on the viability of HUVECs was determined using a CellTiter-Glo^®^ Luminescent Cell Viability assay (Promega Corporation, Madison, WI, USA), as previously described in the cellular viability section. 

### 4.7. HUVEC Spheroids Sprouting Assay

HUVEC spheroids were prepared by firstly staining the cells by incubating 190,000 cells with 2 µM solution of CellTracker^TM^ Green CMFDA Dye (Invitrogen Molecular probes, Paisley, UK) for 30 min in a humidified incubator set at 37 °C and 5% CO_2_, followed by centrifugation for 5 min and the removal of the supernatant. HUVEC pellet was suspended with supplemented HUVEC medium (5 mL) mixed with methocel solution (1.25 mL), which should be prepared earlier [48]. Then, 25 µL of the cell suspension was pipetted onto the cover of the Petri dish. Approximately 50 drops were pipetted in each Petri dish. Finally, drops were kept upside down for 24 h in a humidified incubator set at 37 °C and 5% CO_2_.

Formed spheroids in each dish (~50 spheroids) were collected separately with 1× PBS and centrifuged at 150× *g* for 5 min. In the meantime, collagen I working solution was prepared on ice by gentle mixing of rat tail collagen I stock (1500 µL) (Millipore, MA, USA) with 10× medium 199 (150 µL) (Sigma-Aldrich, Saint Louis, MO, USA) and ice-cold sterile 1N NaOH (34 µL), which turned a red color. Each spheroid pellet was layered with methocel solution, having 4% FBS (0.25 mL), collagen I working solution (0.25 mL) and 60 µL of basal medium or VEGF 30 ng/mL or DCA 25 mM or a combination of both. Immediately after gentle mixing, the mixture was added to a pre-warmed 24-well plate and incubated in a humidified incubator set at 37 °C and 5% CO_2_ for 24 h, allowing for collagen polymerization and spheroid sprouting. After 24 h, spheroids were captured using an inverted microscope with 20× magnification. The sprout length in 12 spheroids in each condition was measured using ImageJ.

### 4.8. Wound Healing Motility Assay

A549 and LNM35 cells were seeded at a density of 1 × 10^6^ cells/well into a 6-well plate. After 24 h, a scratch was made through the confluent monolayer by using a 200 µL tip. After that, the cells were washed twice with 1× PBS followed by the addition of supplemented fresh medium having a drug vehicle or DCA. At the top of the plate, two places were marked for monitoring the decrease in the wound size over time, using an inverted microscope at objective 4× (Olympus 1X71, Tokyo, Japan). The plates were incubated in a humidified atmosphere at 37 °C, and 5% CO_2_ and the wound width was measured at 0, 2, 6 and 24 h after incubation. The migration distance was expressed as the average of the difference between the measurements at time zero and the 2, 6 and 24 h time periods.

### 4.9. Matrigel Invasion Chamber Assay

Following the manufacturer’s protocol (Corning, Bedford, MA, USA), 0.5 mL RPMI-1640 medium, supplemented with 10% FBS, was added to the bottom chambers. After that, cancer cells were seeded at a density of 1 × 10^5^ cells/0.5 mL into the upper chambers in a medium lacking FBS in the presence and absence of DCA. The plate was kept in a humidified incubator at 37 °C and 5% CO_2_ for 24 h. Invasive cells degrade the Matrigel and pass through the 8 µm pores of the insert. The upper chambers’ non-penetrating cells were removed by gently rubbing the area with a cotton swab. Then, the semipermeable membrane was removed using a very fine scissor. The invasive cells were detected using a CellTiter-Glo^®^ Luminescent Cell Viability assay (Promega Corporation, Madison, WI, USA) previously described in the cellular viability section. The effect of DCA on cellular invasion was presented as a percentage (%) by comparing the invading cells in the presence of DCA with the control. 

### 4.10. Statistical Analysis

Apart from the in ovo assay and experiments on nude mice, each experiment was carried out at least three times. Data are expressed as mean ± S.E.M. The statistical analysis was performed using GraphPad Prism version 8.3.1 for Windows (GraphPad Software, San Diego, CA, USA). An unpaired *t*-test was used to assess the difference between two groups. One-way ANOVA followed by Dunnett’s multiple comparison test were used to compare 3 or more groups to the control group. Additionally, a one-way ANOVA followed by Tukey’s multiple comparison test was used for the combination experiments. * *p* < 0.05, ** *p* < 0.01, *** *p* < 0.001, and **** *p* < 0.0001 indicate significant differences.

## Data Availability

Not applicable.
